# Subacute Appendicitis Within a De Garengeot Hernia: A Rare Case and Management Considerations

**DOI:** 10.1155/cris/1549773

**Published:** 2026-02-17

**Authors:** Christopher Rossi, Gabrielle Rossi, Trent Proehl

**Affiliations:** ^1^ Department of Surgery, Hopedale Medical Complex, Hopedale, Illinois, USA

**Keywords:** appendicitis, De Garengeot hernia, femoral hernia

## Abstract

**Introduction:**

De Garengeot hernias are a rare type of femoral hernia which contains the appendix inside the hernia sac. Prompt surgical intervention is required when appendicitis develops within the hernia. Diagnosis and management are often challenging due to the uncommon nature of this condition.

**Case Presentation:**

We report the case of a 61‐year‐old female who presented to the clinic with right lower quadrant pain and an erythematous bulge in the right groin. A CT scan confirmed the presence of a femoral hernia with an incarcerated appendicitis. She was managed by the general surgery team and underwent a staged appendectomy followed by femoral hernia repair via an open anterior approach. The patient recovered uneventfully.

**Discussion:**

De Garengeot hernia is a rare clinical entity which is often misdiagnosed preoperatively. The decision to treat in a one‐ or two‐stage fashion, as well as open or laparoscopically, is nuanced and requires consideration of contamination within the femoral space and if mesh is required for hernia repair. CT imaging can aid in diagnosis but has a relatively low sensitivity and specificity. Prompt recognition and surgical management are critical for preventing complications.

**Conclusion:**

De Garengeot hernia is a rare but serious surgical condition requiring prompt intervention for optimal outcomes.

**Summary**

• This case report describes the challenges of diagnosis and management of a De Garengeot hernia.
• It highlights a unique management approach to an uncommonly encountered clinical scenario.

## 1. Introduction

Femoral hernias are uncommon and represent about 2%–4% of all hernias [1]. Nearly 50% of femoral hernias present with incarceration and 5%–20% with strangulation [2, 3]. De Garengeot hernia is a rare type of femoral hernia which contains the appendix inside the hernia sac. Only about 1% of all femoral hernias contain the appendix, and an estimated 0.08%–0.13% contain an incarcerated acute appendicitis [4, 5]. We present the case of a 61‐year‐old female who presented with right lower quadrant pain and a right groin bulge. Due to the rare occurrence of this type of hernia, questions remain regarding the preferred operative management strategy of such cases.

## 2. Presentation of Case

A 61‐year‐old female presented to her primary care physician with abrupt onset of sharp right lower quadrant pain and a bulge in the right groin that “felt like labor pains.” She first noticed the pain after lifting heavy items 3 days prior. She denied fever, constipation, or other gastrointestinal complaints. Aggravating factors include sitting up straight and straining. No relieving factors identified. Past medical history is significant for Grave’s disease. She had a past surgical history of tubal ligation and endometrial ablation. On physical exam she was found to have a 3–4 cm nonreducible, slightly indurated right groin mass below the inguinal ligament. CT scan was ordered. The outside provider reviewed the results of the CT scan 3 days after performing the study and referred her to general surgery.

Her vital signs at the time presentation were height 5’6", weight 161 lbs, BMI 26.1. Temp 97.3 F, and BP 162/80. Pulse 82, RR, 19 spO2 97%. The physical exam demonstrated a tender, nonreducible, mildly erythematous 3 cm mass in the medial right groin. Her abdomen was nontender. Normoactive bowel sounds. No organomegaly present.

**Figure 1:**
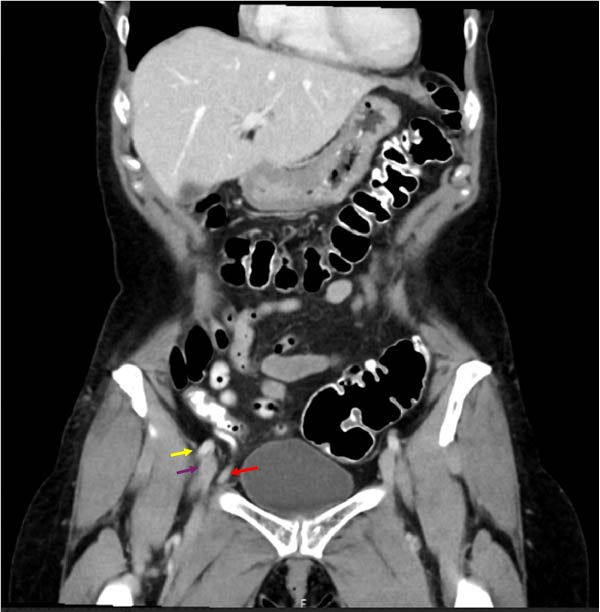
Yellow arrow = femoral artery (coronal), purple arrow = femoral vein (coronal), red arrow = appendix entering femoral canal (coronal).

**Figure 2:**
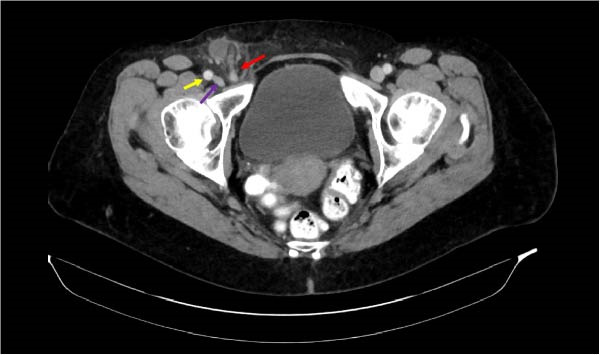
Yellow arrow = femoral artery (axial), purple arrow = femoral vein (axial), red arrow = appendix entering femoral canal (axial).

Abdominal/pelvic CT demonstrated a right femoral hernia containing the tip of the appendix. The appendix was inflamed, consistent with subacute appendicitis (Figures 1 and 2). A 2 cm fluid collection surrounding the tip of the appendix was noted. No intraabdominal fluid or pathology appreciated. She was offered laparoscopic appendectomy with staged femoral hernia repair. Preoperative laboratory data showed WBC 5.4, Hgb 13.1. HCT 39.9. Plt 255. Unremarkable differential. Interestingly, her C‐reactive protein was normal at 2.09 mg/L. Electrolyte panel and liver function tests were unremarkable.

**Figure 3:**
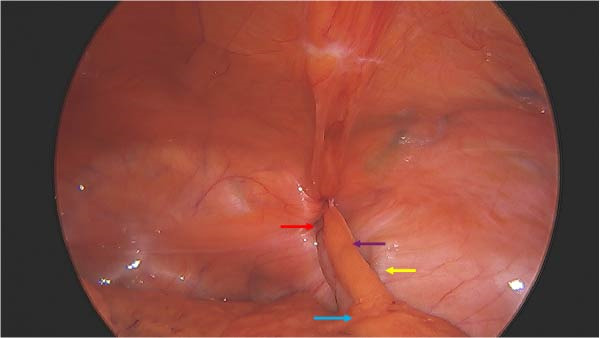
Purple arrow = mesoappendix, red arrow = femoral canal, yellow arrow = external iliac artery and vein, blue arrow = cecum.

The results of the CT scan were reviewed with the patient, and she agreed to proceed with operative intervention. She was taken to the operating room for laparoscopic appendectomy. She was given 1 g ertapenem IV preoperatively, and general anesthesia was induced. Intraoperatively, the distal portion of her appendix was found trapped within the femoral hernia (Figure 3). Gentle dilation of the femoral defect allowed retraction of the appendix out of the defect into the abdominal cavity. The tip of the appendix was noted to be erythematous and partially disrupted. There was no purulence within the femoral canal. The base of the appendix was then resected with a linear stapler. The blood supply to the mesoappendix was taken down in the usual fashion. Next, the appendix was placed in a retrieval bag and removed. The femoral defect was intentionally left open to avoid a closed‐space abscess within the femoral canal. Final pathology revealed foci of chronic inflammation, fibrosis, and surrounding fat necrosis. No carcinoma was identified. The patient recovered uneventfully and was discharged home the same day with plans to return for elective femoral hernia repair via an anterior approach at a later date.

The patient presented to the office 2 weeks post‐op complaining of a 3‐day history of increasing right groin pain and swelling. She denied any fever, chills, or GI complaints, and was tolerating a regular diet. On physical exam, she had normal vitals and was afebrile. She was found to have right groin fullness at the femoral base with slight erythematous skin changes. A palpable, nonreducible mass was appreciated in the right groin. The abdominal exam was unremarkable. A concern for infection within the femoral hernia sac was discussed with the patient. Ultrasound and laboratory analysis were ordered.

CBC was normal. C‐reactive protein was slightly elevated at 8.7 mg/L. Ultrasound of the groin revealed a mildly enlarged 2 cm × 2 cm lymph node but no associated fluid collections or evidence of small bowel loops. She was placed on metronidazole 500 mg PO TID and Levaquin 500 mg PO QD for 10 days and scheduled for short‐term follow‐up. She was seen 1 week later. Right groin evaluation revealed no significant abnormalities other than slight fullness in the femoral space. There was no overlying skin erythema evident.

Patient was reexamined the next week after completing her antibiotics. A physical exam revealed a small 1 cm firmness in the femoral space but no erythema.

1 month later, the patient underwent elective right femoral hernia repair via open anterior approach directly over the preexisting defect. The hernia sac was identified and retracted anteriorly and dissected down to the foramen ovale. A 1 cm defect was seen at the base of the sac. The base of the sac was suture ligated. A primary repair of the hernia defect was performed with two monofilament polypropylene sutures. The wound was then irrigated and closed in two layers. The patient was discharged home without complication. She had an uneventful postoperative course and a normal recovery.

The patient was seen for a 2 week follow‐up visit, and her wound was clean, dry, and well‐approximated, without evidence of infection or recurrent hernia. 1 year later, the patient had no evidence of recurrence or inguinal complaints.

## 3. Discussion

The approach to acute appendicitis within a femoral hernia is debated. The decision between a one‐ and two‐stage procedure is dependent upon the amount of contamination in the femoral space from the inflamed appendix and if mesh would be required for the hernia repair. The majority of femoral hernia defects are small and lend themselves well to primary closure without the use of mesh [6]. Up to 2% of patients with appendicitis may harbor carcinoma on final pathology [7].

We prefer the laparoscopic approach for the appendectomy, which allows for adequate visualization of the base of the appendix to obtain adequate margins in the event that a carcinoma is present within the specimen. In addition, the laparoscopic approach allows for clear visualization of the size of the femoral defect and an assessment of if mesh may be required. We chose a staged femoral hernia repair using an anterior approach. This was done to evaluate for any residual detritus within the hernia sac and also to facilitate management of any infectious process that may have been present. We used a limited 3 cm incision over the femoral defect infra‐inguinally and performed a primary repair after excising the hernia sac.

Choosing an inguinal or femoral approach for the initial procedure can create challenges for ensuring adequate appendiceal stump visualization. For larger femoral defects that may require mesh, staged femoral hernia repair may be a more appropriate approach.

De Garengeot hernia remains a rare clinical entity, with fewer than 500 cases reported in the literature to date, the majority described as individual case reports or small case series. In a recent systematic review of published cases, Guenther et al. [8] identified ~220–230 well‐documented cases, highlighting the heterogeneity of presentation and operative management strategies. The majority of cases occur in women, with over an 80% female predominance reported in this review. The review demonstrated that the most common reported approach involved appendectomy with concomitant hernia repair performed during a single operation, typically through an open groin incision. Both open and laparoscopic appendectomy techniques have been described, with mesh use generally avoided in the presence of appendiceal inflammation or perforation.

Reported outcomes across published cases are generally favorable, though postoperative wound complications, including surgical site infection and abscess formation, remain the most commonly cited adverse events, particularly when hernia repair is performed in a contaminated field. Recurrence rates are inconsistently reported but appear low overall, especially when definitive hernia repair is delayed until resolution of inflammation [8]. Importantly, several authors advocate a staged approach in cases of appendicitis or local contamination, citing concern for infectious complications associated with immediate femoral canal closure [9, 10].

Our management strategy aligns with these findings. We elected to perform laparoscopic appendectomy with delayed femoral hernia repair to minimize contamination of the femoral canal and avoid mesh placement in an inflamed field. The femoral defect was intentionally left unrepaired at the index operation to prevent formation of a closed infected space, a complication described in prior reports. Definitive open anterior femoral hernia repair was subsequently performed once local inflammation had resolved, allowing for safe primary repair. The patient experienced no wound infection, no recurrence on serial examinations, and remained asymptomatic at 1‐year follow‐up. Compared with prior series summarized in the systematic review, our approach reflects a conservative, staged strategy that prioritizes infection control while achieving durable hernia repair with favorable short‐ and medium‐term outcomes.

## 4. Conclusion

De Garengeot hernia is a rare clinical entity that requires timely surgical intervention for optimal outcomes. Early recognition and definitive management are necessary for mitigating patient risk.

## Author Contributions

Data collection and preparation of the manuscript: Christopher Rossi. Preparation of the manuscript: Gabrielle Rossi. Management of the patient and Guarantor: Dr. Trent Proehl.

## Funding

This case report did not receive any form of funding or grant from public, private, or nonprofit organizations.

## Ethics Statement

In accordance with our institution’s policy, case reports do not require Institutional Review Board (IRB) approval, provided a written informed consent has been obtained from the patient.

## Consent

A written informed consent was obtained from the patient for publication of this case report and accompanying images.

## Conflicts of Interest

The authors declare no conflicts of interest.

## Data Availability

The data that support the findings of this study are available from the corresponding author upon reasonable request.
